# Supplementary material for: Automatic monitoring of neural activity with single-cell resolution in behaving Hydra
Source: Sci Rep. 2024 Mar 1;14:5083. doi: 10.1038/s41598-024-55608-2 (PMC10907378; doi:10.1038/s41598-024-55608-2)
Supplement: Supplementary file 1 — Supplementary Information. [file 41598_2024_55608_MOESM1_ESM.docx]

**SUPPLEMENTAL DATA**


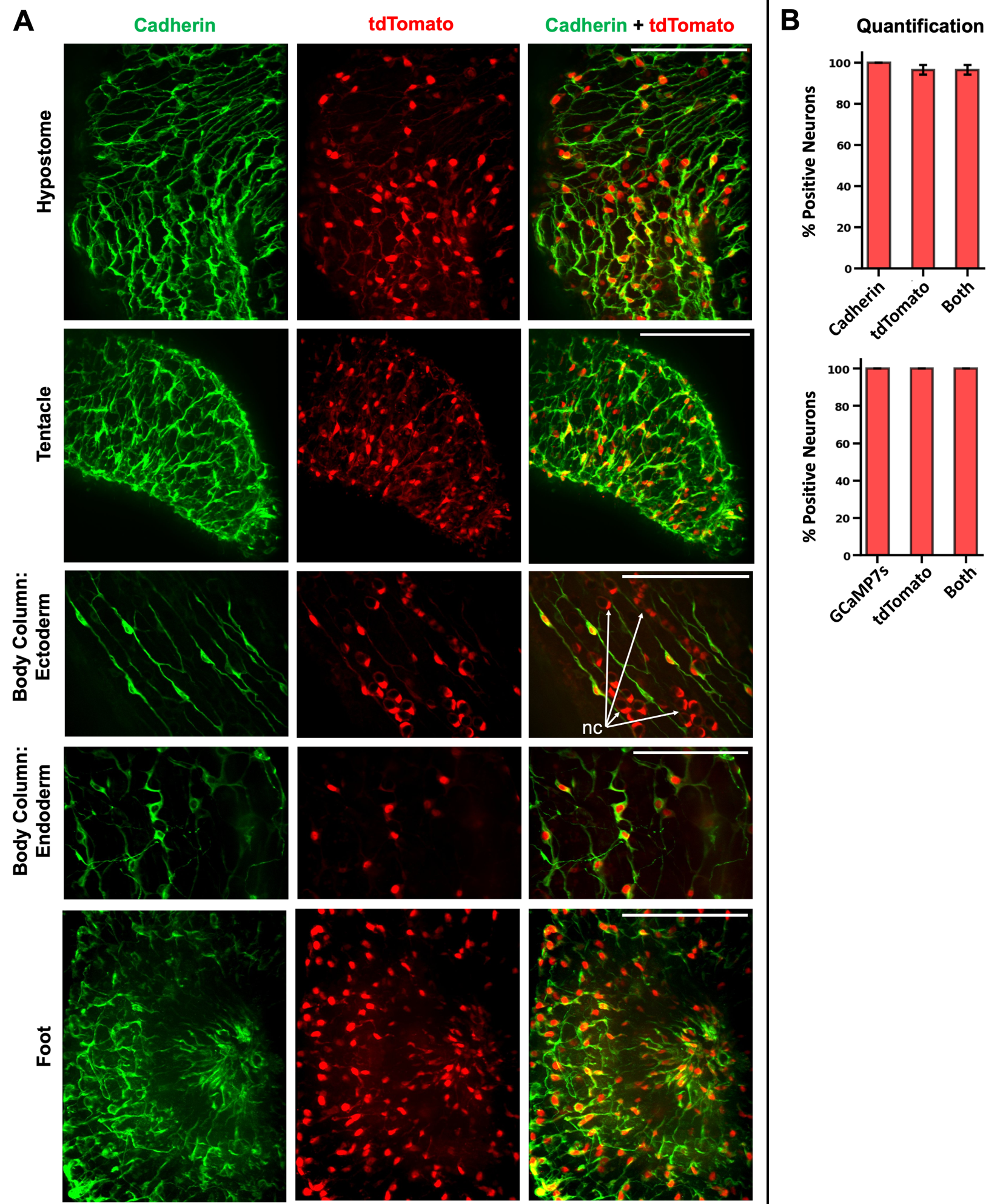


**Figure S1. Percentage of dually labeled transgenic neurons. Related to Figure 2.**  (A) Immunostaining of neurons with anti-*Hydra* cadherin antibody (left), anti-tdTomato antibody (middle), and overlap of the two stains (right). Different *Hydra* body parts are labeled on the left. Nematocytes (nc) are indicated with white arrows. The confocal images are maximum intensity projections of short stacks (5-20 μm) through each body region. Scale bars: 100 μm. (B) The percentage of neurons stained with both anti-*Hydra* cadherin and anti-tdTomato are shown (top). The percentage of cytoplasmic GCaMP7s expressing neurons that also express nuclear tdTomato are shown (bottom) (mean $\pm$ std, n = 5 animals).
